# Population Hemoglobin Mean and Anemia Prevalence in Papua New Guinea: New Metrics for Defining Malaria Endemicity?

**DOI:** 10.1371/journal.pone.0009375

**Published:** 2010-02-24

**Authors:** Nicolas Senn, Seri Maraga, Albert Sie, Stephen J. Rogerson, John C. Reeder, Peter Siba, Ivo Mueller

**Affiliations:** 1 Papua New Guinea Institute of Medical Research, Goroka, Papua New Guinea; 2 Department of Medicine, University of Melbourne, Melbourne, Australia; 3 Swiss Tropical Institute, Basel, Switzerland; 4 Burnet Institute, Melbourne, Australia; London School of Hygiene and Tropical Medicine, United Kingdom

## Abstract

**Background:**

The hypothesis is that hemoglobin-based metrics are useful tools for estimating malaria endemicity and for monitoring malaria control strategies. The aim of this study is to compare population hemoglobin mean and anemia prevalence to established indicators of malaria endemicity, including parasite rates, rates of enlarged spleens in children, and records of (presumptive) malaria diagnosis among populations living with different levels of malaria transmission.

**Methodology/Principal Findings:**

Convenience sample, multisite cross-sectional household surveys conducted in Papua New Guinea. Correlations (r^2^) between population Hb mean and anemia prevalence and altitude, parasite rate, and spleen rate were investigated in children ages 2 to 10 years, and in the general population; 21,664 individuals from 156 different communities were surveyed. Altitude ranged from 5 to 2120 meters. In young children, correlations between altitude and parasite rate, population Hb mean, anemia prevalence, and spleen rate were high (r^2^: −0.77, 0.73, −0.81, and −0.68; p<0.001). In the general population, correlations between altitude and population Hb mean and anemia prevalence were 0.83 and 0.85, respectively. Among young children, parasite rate correlated highly with anemia prevalence, population Hb mean, and spleen rate (r^2^: 0.81, −0.81, and 0.86; p<0.001). Population Hb mean (corrected for direct altitude effects) increased with altitude, from 10.5 g/dl at <500 m to 12.8 g/dl at >1500 m (p<0.001).

**Conclusions/Significance:**

In PNG, where Plasmodium vivax accounts for an important part of all malaria infections, population hemoglobin mean and anemia prevalence correlate well with altitude, parasite, and spleen rates. Hb measurement is simple and affordable, and may be a useful new tool, alone or in association with other metrics, for estimating malaria endemicity and monitoring effectiveness of malaria control programs. Further prospective studies in areas with different malaria epidemiology and different factors contributing to the burden of anemia are warranted to investigate the usefulness of Hb metrics in monitoring malaria transmission intensity.

## Introduction

Malaria, with about 500 million cases reported per year worldwide, remains one of the predominant diseases in tropical countries. The intensity of malaria transmission depends on a variety of factors such as climate and geographical context, human population, socio-economical level and vector ecology, as well as the type of control strategies in place. Nowadays, several efficient strategies are being used to decrease the malaria burden. Most prominent among them are insecticide treated bed nets (ITN), indoors residual spraying (IRS), artemisinin combination treatments (ACT)[1], [2], [3] and intermittent preventive treatment strategies in pregnant women or infants[4]. Adequately and simply measuring malaria endemicity is thus of prime importance in order to plan and monitor effectiveness of these control interventions.

Different tools and metrics have been used for more than 150 years to investigate the level of malaria endemicity in a given population. Traditionally, the spleen rate (SR), i.e. the prevalence of splenomegaly in a population, has been used to classify malarial endemicity[5]. This measure however suffers from a lack of specificity. Indeed, many diseases highly prevalent in the tropics may also lead to splenomegaly like visceral leishmaniasis or schistosomiasis. More importantly, it has been shown in Papua New Guinea (PNG) by Brabin *et al*. that the spleen rate may vary between sub-populations of non-pregnant women with similar levels of exposure to malaria[6], probably due to differences in immune response to malaria. Splenomegaly can also be difficult to measure reproducibly.

In the 1950's, the prevalence of blood stage malaria parasites (parasite rate for all species, PR) was introduced as the principal metric for monitoring malarial endemicity and remains the method of choice[7]. This metric is much more specific but tends to be influenced by seasonal fluctuations and is strongly age-dependent. In addition, it requires blood collection and expert microscopy that may not be available in resource-limited settings. Utilizing both SR and PR in children 2–10 years, a classification of malaria endemicity was proposed by WHO and revised by Metselaar *et al* [7]: hypoendemic if SR 2–10 or PR 2–10 is <10%, mesoendemic if SR 2–10 or PR 2–10 is 10–50%, hyperendemic if SR 2–10 or PR 2–10 is 51–75% and holoendemic if SR 2–10 or PR in children <1year is >75%. These measures may provide good estimates of malaria endemicity in areas where malaria is highly prevalent, but they are less accurate when malaria transmission is lower[8] such as in an aggressive control program. The Annual Parasite Incidence (API = number of malaria cases/population, usually expressed per 1000, a malaria episode is confirmed by a positive blood slide) is thus often used in areas of lower transmission. However, this metric requires even more resources than measuring PR including a reliable reporting system for malarial episodes.

All these different metrics have been integrated into control strategies such as the Global Malaria Eradication Programme coordinated by the World Health Organization (WHO) since the mid 1950's. More recently the Malaria Atlas Project (www.map.ox.ac.uk), used the *Plasmodium falciparum* (*Pf*) parasite Rate (*Pf*PR), usually in children 2–10 years[9], and *Pf* Annual Parasite Incidence (*Pf*API) to map *Pf* endemicity. As part of this work, Hay and colleagues published an extensive and detailed review on the tools and strategies available to monitor malaria endemicity [10].

Given the limitations of the traditionally used measures of malaria endemicity outlined above, there is therefore a need to identify additional easily implementable tools for the accurate measurement of malaria endemicity in a variety of transmission settings.

It is well known that one of the main clinical features of malaria at an individual level is a fall in hemoglobin (Hb), often resulting in anemia [11], [12], [13]. However, little is known of how Hb varies at a population level relative to changes in malaria transmission intensity. Two studies have shown associations between altitude variations and changes in malaria prevalence, with *Pf* prevalence decreasing with altitude[14], [15]. In the same studies, Hb concentrations of individuals were also found to increase with altitude. This suggests that both the population hemoglobin mean (popHb) and anemia prevalence (AP), following correction for altitude, correlate directly with the variations of malaria transmission intensity and may thus be useful as measures of malaria endemicity. However, the evidence for this direct correlation is lacking as the existing data are limited by the sample size or the number of locations. Furthermore, all studies have been performed in Africa where *Pf* is the predominant species. Therefore, there is a need to investigate the association between Hb measurements at a population level and malaria in larger datasets from areas with a wider range of altitude, malaria prevalence and plasmodium species.

PNG is situated in the South-West Pacific and presents a wide range of malaria endemicity due to its mountainous geography, ranging from highly prevalent at sea level to absent in the highlands (up to 4000 meters)[16]. Another characteristic of PNG is that all four species of plasmodium are present, with *Plasmodium falciparum* (*Pf*) responsible for more than 50% of all clinical malaria episodes, and *Plasmodium vivax (Pv)* being responsible for most of the remaining cases. In past surveys, entomological inoculation rate (EIR) has varied between <1 and 400[16]. Overall, malaria is the main cause of hospital admissions in lowland areas, but is absent in much of the highlands. Thus, PNG is an ideal site to investigate the relationship and correlation between classical measures of malaria transmission intensity, altitude and variations of population Hb levels and anemia prevalence.

## Methods

### Study Site and Patients

Individual informed consent was obtained from all participants or in case of children their guardians before enrolment into the study. Malaria surveys conducted in the Highlands of PNG [17], [18], [19], [20] were performed on behalf of the PNG National Department of Health and the consent was verbal. In the lowlands surveys that were parts of research studies, written consent was obtained. All participants specifically consented for a finger prick blood sample to be collected and assessed for the presence of malaria and anaemia. This Consent process was approved by the PNG Medical Research Advisory Council of Papua New Guinea (respective approval numbers are: Highlands: 00.26, Sepik: 05.08 and Madang: 05.20)

The study was performed in PNG alongside a series of household malaria surveys performed in three different regions of the country: the Highlands (inland, 500–2100 m), the Middle Sepik (lowland, <500 m) and Madang (coastal, <500 m). The different villages were chosen according to their location and representativeness in terms of presumptive malaria endemicity and population diversity. Further details of the selection of Highlands and Sepik survey locations and of the survey methodology are given by Mueller *et al*. (2003–2009) [17], [18], [19], [20], [21], [22], [23], [24]. In short, within each village a household based survey was conducted using convenience sampling. All members of a participating household were enrolled in the surveys after individual consent. Medical history of malaria (history of fever, treatment for malaria and other spontaneously reported symptoms) and a brief medical examination including spleen measurement (using Hackett's classification), axillary temperature and targeted clinical examination were performed. All patients presenting with signs or symptoms of malaria had a rapid diagnostic test performed, and malaria treatment was provided if positive. A finger prick was performed on each participant, blood slides with thick and thin films for malaria microscopy were made and Hb was measured using a portable Hemocue 201® machine (Angelholm, Sweden) in g/dl. The Madang surveys were conducted in the context of preparatory works for a trial of intermittent preventive treatment in infants (IPTi), using the same methodology. Surveys conducted in villages with epidemic outbreaks of malaria were not used for this study.

All blood smears were stained with 2.5% buffered Giemsa (pH 7.2) for 35 minutes and examined by light microscopy. Slides were declared negative if no parasites were seen in 100 thick film fields by two different microscopists. The parasite species in positive films were identified and densities were recorded as the number of parasites/200 WBC. Densities were calculated assuming 8,000 WBC/µl [25]. All slides with densities less than 200 /µl, along with a randomly selected 20% of all blood films were routinely re-examined. If less than 80% concordance was achieved between evaluations, the entire batch of slides was re-read.

There is no standardized cut-off value for defining anemia for individuals [26] in tropical countries. In order to calculate the prevalence of anemia (AP) for each community, we defined a cut-off value of 11 g/dl which is clinically meaningful, easy to interpret from a public health perspective and gives a good proxy to anemia (see Supplementary Material S1). Crude and adjusted population hemoglobin mean values (popHb) were calculated for all communities. Adjustment was done for sex, age and altitude. Sex and age were adjusted using non-parametric regression splines. Hb was adjusted for altitude using the model published by the CDC [27], [28], [29] with the following formula calculating the correcting factor for the different Hb values: Δ*Hb [g/L] = −0.32 x (altitude[m] x 0.0033)+0.22 x (altitude[m] x 0.0033)^2^*.

All metrics (popHb, AP, SR and PR) were calculated for both the general population and children 2–10 years. Indeed, spleen rate and parasite rate are usually calculated in this age group for defining malaria endemicity categories[7].

### Statistical Methods

All data were analyzed using STATA software, version 10.0. Correlations between the different metrics and altitude were investigated by calculating Pearson's correlation coefficients (r^2^); significance was assessed by calculating p-values. The same software was used to draw a graph of the correlations between the different metrics weighted for the number of individuals per village. We performed also an ANOVA analysis to investigate the variation of the different metrics across altitude strata. As both Hb metrics and prevalence of infection are subject to measurement and sampling errors, major axis regression [30] was used to determine the symmetric relationship between Hb metrics and prevalence of infection in children 2–10 yrs. The resulting relationships are bi-directional, i.e. prevalence of infection can be predicted from Hb metrics and vice versa All analyses were done on data aggregated at the village/population level and did not include measures of individual level variation.

## Results

21,664 individuals from 156 different communities were surveyed from the Highlands, Madang and Sepik areas, altitudes ranged from 5 to 2120m above sea level. On average there were 143 participants in a village (range [35–387]). Overall, the median for age was 14.9 years (range [0–99]) with 51.4% of participants female. Among the villages, 27 were at elevations below 500 m, 17 were between 500 and 999 m, 55 were between 1000 and 1499 m and 57 were above 1500 m altitude. The ratio of Pf/Pv prevalence rates among the 145 villages is 2.04 (range [0–17]).

The PR in 2–10 (F_3, 148_ = 75.6, p<0.001) and SR 2–10 (F_3,147_ = 47.0, p<0.001) were found to decrease significantly from 58.1% and 37.8% in villages <500 m to 4.3% and 1.6% in those >1500 m. AP also decreased significantly with altitude (<500 m: 62.8%, >1500 m: 12.3%, F_3,143_ = 114.0, p<0.001) while PopHb increased (altitude adjusted: <500 m 10.5 g/dl, >1500 m 12.8 g/dl, F_3,148_ = 74.3, p<0.001). Complete altitude stratification data are shown in Table 1.

**Table 1:** Metrics for village malaria endemicity by altitude.

Altitude		<500 m			500–999 m			1000–1499 m			>1500 m		
		n = 27			n = 17			n = 55			n = 57		p
	Mean	CI95%	range	Mean	CI95%	range	Mean	CI95%	range	Mean	CI95%	range	
PopHb (crude)	**10.5**	10.4–10.7	9.0–11.3	**11.2**	10.9–11.6	10.0–12.9	**12.4**	12.2–12.6	10.5–14.4	**13.3**	13.1–13.4	11.7–14.7	<0.001
PopHb Adj*	**10.5**	10.4–10.7	9.0–11.3	**11.2**	10.8–11.5	10.0–12.8	**12.2**	11.9–12.4	10.2–14.1	**12.8**	10.4–10.7	11.4–14.2	<0.001
AP	**0.63**	0.60–0.66	0.52–0.82	**0.46**	0.39–0.53	0.17–0.68	**0.25**	0.21–0.29	0.01–0.58	**0.12**	0.1–0.14	0–0.35	<0.001
SR 2–10	**0.38**	0.31–0.44	0–0.6	**0.36**	0.23–0.49	0–0.75	**0.12**	0.08–0.16	0–0.68	**0.02**	0.01–0.02	0–0.14	<0.001
PR 2–10	**0.58**	0.51–0.65	0.1–0.92	**0.41**	0.30–0.51	0.05–0.69	**0.2**	0.15–0.25	0.0–0.68	**0.04**	0.03–0.06	0.0–0.21	<0.001

*adjusted for altitude.

AP = anemia prevalence, SR 2–10 = spleen rate in children 2 to 10 years and PR 2–10 = parasite rate in children 2 to 10 years.

**Figure 1:**
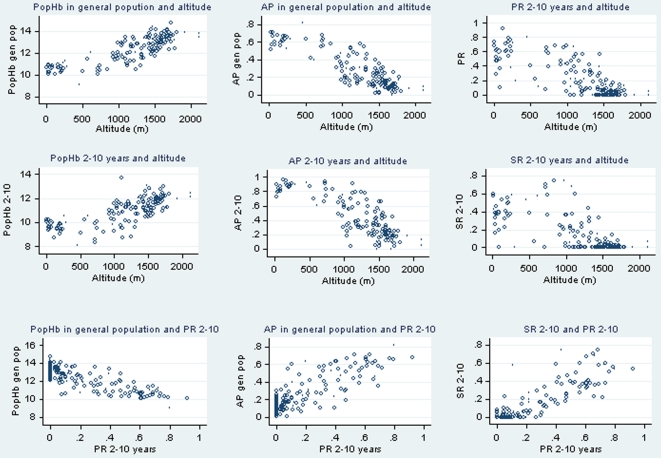
Scatter plots of correlations with altitude and Parasite Rate 2–10 years for different metrics. The different metrics are: Population hemoglobin mean (PopHb), Anemia Prevalence (AP), both in general population and 2–10 years and Spleen Rate in 2–10 years (SR 2–10). Each circle represents one village.

Overall, very high correlations were observed between altitude and the different metrics for malaria endemicity, as shown in Table 2, both in the general population: PR = −0.80, SR = −0.60, crude popHb = 0.83 and AP = −0.85 and in children 2–10 years: PR 2–10 = −0.77, SR 2–10 = −0.68, crude popHb 2–10 = 0.73 and AP 2–10 = −0.81 (all correlations: n = 156, p<0.001). Similarly high correlations were found between PR (overall and in children 2–10) and other metrics (Table 2, p<0.001). Plots of individual measures however do reveal differences between the different measures (Figure 1). PopHb and AR decrease linearly at altitudes above 500 m but do vary less at lower altitudes, while at altitude >1500 m rates of enlarged spleens were very low. Accordingly, the strength of the association between the different metrics varied across altitudinal strata (Table 3). The correlations between Hb metrics (popHb and AP) and PR 2–10 were strongest between 1000–1499 m altitude, and disappeared above 1500 m: r^2^<500 m: −0.42 & 0.48, 500–999 m: −0.53 & 0.43, 1000–1499 m: −0.62 & 0.68 and > = 1500 m: −0.14 & 0.2.

**Table 2:** Summary of Pearson's coefficients of correlation (r^2^) between altitude (Alti), population haemoglobin mean (PopHb) crude or adjusted (for age, sex and altitude), anaemia prevalence (AP), parasite rate (PR) and spleen rate (SR) in the general population (Pop) and children 2–10 years, in non-epidemic context*.

		Crude PopHb (pop)	Crude PopHb (2–10 y)	Adjusted PopHb	AP (pop)	AP (2–10 y)	PR (pop)	PR (2–10 y)	SR (pop)	SR (2–10 y)
**Alti**		0.83	0.73	0.78	−0.85	−0.81	−0.8	−0.77	−0.6	−0.68
**Crude PopHb**	Pop		0.94	0.99	−0.98	−0.95	−0.79	−0.81	−0.67	−0.75
	2–10 years			0.95	−0.93	−0.95	−0.7	−0.77	−0.65	−0.75
**Adjusted PopHb**	Pop				−0.97	−0.94	−0.77	−0.79	−0.65	−0.74
**AP**	Pop					0.96	0.79	0.81	0.67	0.76
	2–10 years						0.75	0.79	0.65	0.74
**PR**	Pop							0.96	0.71	0.82
	2–10 years								0.76	0.86
**SR**	Pop									0.93
	2–10 years									

*All coeficients have a p<0.001.

AP = anemia prevalence, SR = spleen rate and PR = parasite rate.

**Table 3:** Correlations between different measures of malaria endemicity by altitudinal strata. AP = anemia prevalence and PR 2–10 = parasite rate in children 2 to 10 years.

Altitude		<500 m			500–999 m			1000–1499 m			> = 1500 m	
		n = 154			n = 147			n = 151			n = 152	
	HbPop	AR	SR 2–10	HbPop	AR	SR 2–10	HbPop	AR	SR 2–10	HbPop	AR	SR 2–10
PR 2–10	−0.42**	0.48**	0.83*	−0.53**	0.43***	0.55**	−0.62*	0.68*	0.32*	−0.14***	0.2***	0.32**
HbPop		−0.93*	−0.29***		−0.96*	−0.52**		−0.96*	−0.6*		−0.9*	−0.19***
AR			0.52**			0.62*			0.65*			0.18***

*p<0.01,

**p<0.05,

***p>0.05.

Using major axis regression, the following relationships were found to best describe the (linear) relationship between Hb metrics and the prevalence of infections in children 2 to 10 years and Hb metrics: P_2to10_ = 0.854 * AP – 0.162 and P_2to10_ = −0.204 * PopHB +2.73.

## Discussion

Using a very large data set from Papua New Guinea, this study has investigated the correlation between altitude, commonly used metrics (spleen rate and parasite rate in children 2–10 years and records of febrile illnesses) and hemoglobin levels expressed as population mean hemoglobin (PopHb) and prevalence of anemia (AP) per community. As shown in earlier studies in Tanzania and Kenya [14], [15], in PNG PR and SR were strongly correlated with altitude indicating that altitude is a reasonable proxy for malaria endemicity in PNG. In the present study, it was observed that both PopHb and AP were also strongly associated with altitude variations. Crude measures of Hb performed as well as those that were adjusted for effects of age, gender and altitude. Hb measurements were also closely correlated with PR and SR.

In an altitude-stratified analysis, a progressive increase of PopHb is observed, from 10.5 g/dl on average below 500 m, to 12.8 g/dl above 1500 m, after correction for altitude. Of factors other than altitude itself which may explain these variations, malaria is likely to be the most important one. Other “prevalence” metrics (AP, SR 2–10 and PR 2–10) all show progressive decreases with increasing altitude. Correlation of Hb metrics and PR 2–10 are stronger at altitude between 500 and 1500 m, where endemicity is higher (see Figure 1 and Table 3).

By nature, single cross-sectional surveys are not well suited to describe malaria endemicity in areas of significant seasonality. Although Hb level also show some seasonal variations [31], they are more strongly associated with the history of parasitaemia preceding the Hb measurement than with concurrent parasite densities [32]. Mean Hb levels thus are a measure of mean exposure in the preceding 3 months rather than representing a single time point like parasite prevalence rate. Nevertheless, when comparing Hb metrics between areas or over the time of an antimalarial intervention, it is important to either conduct surveys during the same period of the year or to adjust for seasonal variations in the relationship between malaria and Hb levels.

Throughout PNG *P. falciparum*, *P. vivax*, *P. malariae* and *P. ovale* are co-endemic. In most areas *P. falciparum* is the most common infection followed by *P. vivax* and in particular in highly endemic areas mixed species infection are very common [24], [33]. While infections with any species can result in anemia, *P. falciparum* infection tend to be associated with a stronger reduction in Hb levels [21], [22], [23] and the higher risk of severe anemia [34], [35] than *P. vivax*. However, as prevalence of infection with all species increases in parallel with increasing endemicity, it is difficult to attribute parts of the observed effect of malaria on Hb levels to individual species. Consequently, overall prevalence rates show stronger association with Hb metrics than either *P. falciparum* or *P. vivax* specific prevalence rates (data not shown). Finally, it is likely that increasing drug resistance to malaria infections will not have the same effect on the different metrics. A recent study in the Gambia showed higher rates of severe anemia in children infected with chloroquine resistant *P. falciparum* [36]. Similarly, the high rates of severe anemia observed in both hospitalized *P. falciparum* and *P. vivax* patients in Indonesian Papua was linked to the very high rate of drug resistance [37]. It is thus possible that that an increase in drug resistance might change the pattern of clinical malaria episodes and that consequently Hb levels might be affected differently from PR.

Besides malarial infections there are a number of different factors that also impact on Hb levels. Of these, it is straightforward to control for altitude, sex and age. As shown in this study, such adjustment may however not necessarily improve the strength of the association between Hb and malarial endemicity.

Other important contributors to the burden of anemia include infections, nutrition status and host genetic traits such as common red blood cell polymorphisms. Among infections, hookworm infestation is probably the most important in PNG. It is highly prevalent throughout the country [38], [39]. Although high egg loads may be associated with anemia in some populations of PNG[40], other studies did not find such a link [38]. Adjusting for the presence of helminth infections would thus potentially improve the accuracy of Hb metric. Unfortunately these data are not available for the PNG surveys. Given the nation-wide presence of hookworm and the lack of consensus on its impact on Hb levels in PNG it is however unlikely that this would have substantially changes the observed relationships between Hb and malaria at the population level. Parvovirus B19 infection has recently been identified as a significant risk factor for severe anemia in PNG[34], but as an infection of young children with limited variation between populations[41] the effect of B19 infection will mainly affect this age group and to a lesser extent the adult population. To-date, no studies have investigated the overall contribution of B19 infections to the burden of anemia. Schistosomiasis, which contributes a lot to the burden of anemia elsewhere, is not present in PNG. In other malaria endemic areas like Africa, it will however be necessary to take into account other infectious agent. In particular, Schistosomiasis as an important confounder and adjustment might thus be required where it is endemic. In conclusion, whereas co-infections might potentially affect population Hb levels, the actual impact will depend on epidemiological context and due to the lack of appropriate co-infection data is often difficult to quantify.

Secondly, dietary factors such as malnutrition or micronutrient deficiencies impact on Hb levels. A recent review by Metz has shown that folate and vitamin B12 deficiency, even though very frequent in developing countries, do not significantly affect the prevalence of anemia[42]. Vitamin A (by affecting erythropoiesis[43]) and Iron deficiencies are the two micronutrients which are contributing the most to anemia. Low levels of Vitamin A may also contribute to an increase risk of malaria [44]. In the 2005 PNG National Malnutrition survey 15% of children <5 years had low levels of VitA with little regional difference. VitA deficiency was however almost completely absent in adults (Saweri, pers. communication). In the same survey 23% of people had elevated transferrin receptor (TfR) levels that were indicative of iron deficiency. Although the prevalence of elevated TfR varied significant with age and among regions, the prevalence of elevated TfR among anemic participants (defined as <10 g/dl for children <11 for adults) was similar in children and adults (42–47%), albeit lower in highlands (19–32%) compared to lowlands population (38–64%). While chronic protein energy malnutrition and resulting growth stunting is highly prevalent throughout PNG [45], acute malnutrition is less common overall but more important in the coastal areas than in the highlands [46]. The relationship between protein-energy malnutrition, malaria and anemia is complex and not fully understood. Some recently published data suggest that malnutrition contributes to both malaria associated morbidity and anemia [47], [48], [49] while an earlier study in lowlands PNG found a reduced risk of malaria in stunted children [50]. However, in studies in highly endemic areas where both have been assessed malaria is usually a much stronger contributor to anemia than malnutrition[51]. Whereas adjusting for nutritional status might thus improve the accuracy and predictive value of Hb metrics, such data is rarely collected in malariological surveys.

Finally, there are a number of genetic traits that are known to impact on hemoglobin levels. By far the most important one in PNG is alpha-thalassemia 2 (-α) which is very common in lowlands PNG but virtually absent in highlands populations[52]. This form of alpha-thalassemia only causes mild symptoms and even in its homozygote presentation, it has only a minor effect on hemoglobin levels[53]. The homozygote however has a significant protection against severe malaria (including severe malarial anaemia) and severe non-malarial illnesses[54]. Thus, a potential negative effect of alpha-thalassaemia on population Hb may be off-set by a reduction in risk of malaria associated (severe) anemia. Its overall impact on popHb and AR is likely to be limited. The other two common red blood cell polymorphisms are South East Asia Ovalocytosis[55] and the Gerbich blood group[56]. Although both traits are restricted to malaria endemic areas[57], they both cause little or no difference in Hb levels[58]. Sickle cell anemia is not present in PNG. Consequently, the impact of host genetic polymorphisms on the association between Hb levels or anemia prevalence and malaria exposure is likely to be minor in PNG. Importantly, changes in malaria transmission intensity will occur much more rapidly than those in frequency of host genetic polymorphisms. Thus, even if such traits may affect Hb levels, changes in popHb or AR over time would still correctly reflect changes in malaria endemicity.

After consideration of these important potential confounders, the correlations between Hb metrics and altitude, parasite prevalence and spleen rates appear to remain robust. This study therefore provides good evidence that popHb and AP are valuable metrics to estimate the burden of malaria in PNG even without adjusting for potential confounders. Before applying them to other settings, in particular those where schistosomiasis is highly prevalent, it will be important to cautiously consider the impact of such confounders when interpreting Hb levels as malaria metics.

The major advantages of using Hb metrics as measures for malaria endemicity is that they require limited resources to be recorded, they are immediately available in the field and are more reproducible than estimating spleen rate. In addition, popHb is very easy to calculate and AP easy to interpret. Last but not least they also provide important additional information on the health status of a population.

Hb metrics thus appear to be promising alternative tools for measuring malaria burden, on their own or in combination with other traditional or more recent serological[59] measures of malaria endemicity, even in areas of high prevalence of non-falciparum infections. They may be particularly useful in areas of moderate to low transmission and could therefore be of best use in public health when monitoring malaria control interventions programs during the preparatory and attack phases[10]. Additional in-depth studies are warranted to better characterize the relationship between Hb levels and malaria transmission and determine the optimal use as new malaria metrics. In addition, prospective studies in areas with different malaria epidemiology and in the context of malarial control initiatives are required. The examination of changes in Hb-based indices over time in populations benefitting from a major anti-malaria intervention would comprise an important test of the usefulness of Hb metrics based endemicity monitoring in the context of rapidly changing malarial epidemiology in Papua New Guinea or elsewhere.

## Supporting Information

Supplementary Material S1 How to define the best Hb cutoff for anemia prevalence?
(0.03 MB DOC)
