# Supplementary material for: Population Hemoglobin Mean and Anemia Prevalence in Papua New Guinea: New Metrics for Defining Malaria Endemicity?
Source: PLoS One. 2010 Feb 24;5(2):e9375. doi: 10.1371/journal.pone.0009375 (PMC2827550; doi:10.1371/journal.pone.0009375)
Supplement: Supplementary Material S1 — How to define the best Hb cutoff for anemia prevalence? (0.03 MB DOC) [file pone.0009375.s001.doc]

# Supplementary material

**How to define the best Hb cut-off for anemia prevalence?**

As mentioned in the main article, there is no standardized cut-off value for defining anemia for individuals in tropical countries. For our main analyses, we used a cut-off value of 11g/dl, as we wanted to choose a value that was clinically meaningful and easy to interpret from a public health perspective. In addition, by using a higher threshold for anemia we detect more cases of anemia thus increasing precision and/or reducing the required smaller sample sizes for individual surveys. For example, in our entire population, the overall prevalence of Hb < 9g/dl is only 8%, while it is almost 30% for Hb<11g/dl. Using a higher cut-off, both reduce the logistical costs of conducting field surveys, in particular when malaria (and anemia) becomes less prevalent in the context of increasing control. The performance of anemia prevalence as a metric of malaria endemicity is little affected by the choice of anemia cut-off. All cut-offs from 9g/dl to 11g/dl do show comparable associations with traditional metric and altitude (Table S1).

**Table S1: Correlations between anemia prevalence (AP) and other traditional metrics and altitude for different Hb cut-offs values**

| **Hb cut-offs for AP** |  | **Altitude** | **PR 2- 10** | **SR 2 - 10** | **% defined as anemic**  (total Pop =21664  2 - 10yrs: 5284) |
| --- | --- | --- | --- | --- | --- |
| 9 g/dl | Pop | -0.68 | 0.78 | 0.78 | 8% |
|  | 2 - 10 years | -0.63 | 0.78 | 0.8 | 15% |
| 10 g/dl | Pop | -0.82 | 0.81 | 0.79 | 17% |
|  | 2 - 10 years | -0.77 | 0.82 | 0.79 | 29% |
| 11 g/dl | Pop | -0.85 | 0.81 | 0.76 | 29% |
|  | 2 - 10 years | -0.82 | 0.79 | 0.74 | 48% |
| all p-values < 0.001 | | | | | |
